# A decennial cross-sectional review of assisted reproductive technology in a Tertiary Hospital in Southwest Nigeria

**DOI:** 10.1186/s12884-023-05964-0

**Published:** 2023-09-20

**Authors:** Tawaqualit Abimbola Ottun, Adeniyi Abiodun Adewunmi, Faosat Olayiwola Jinadu, Ayokunle Moses Olumodeji, Fatimat Motunrayo Akinlusi, Kabiru Afolarin Rabiu, Oluwarotimi Ireti Akinola, Adetokunbo Olusegun Fabamwo

**Affiliations:** 1 Department of Obstetrics and Gynaecology, Lagos State University College of Medicine & Teaching Hospital, Lagos, Nigeria; 2 Department of Radiology, Lagos State University Teaching Hospital, Lagos, Nigeria; 3 Department of Obstetrics and Gynaecology, Lagos State University Teaching Hospital, Lagos, Nigeria

**Keywords:** In-vitro fertilization, Intra-cytoplasmic sperm injection, Artificial reproductive technology, Infertility, Tertiary Hospital, Southwest Nigeria

## Abstract

**Background:**

The World Health Organization recommends that Assisted Reproductive Technology be complementary to other ethically acceptable solutions to infertility. Whereas fertility centres are increasing in number in urban regions of Africa, published reports of their performance are sparse. We present a 10-year review of assisted reproductive technology performed in a public tertiary centre in Lagos, Nigeria.

**Methods:**

This was a hospital-based, retrospective, cross-sectional review of 604 women, over a 10-year period that had in-vitro fertilization or in-vitro fertilization with intra-cytoplasmic sperm injection at the Institute of Fertility Medicine, Lagos State University Teaching Hospital. Data obtained were expressed in descriptive statistics and Pearson correlation was used to determine the strength of linear relationship between two continuous variables at a significance level of *p* < 0.05.

**Results:**

The mean age of the women was of 37.7 ± 6.2 years and 89.7% had no previous parous experience. About 27.2% of the male partners had normal seminal fluid parameters while 4.6% had azoospermia. Median serum follicle stimulating hormone of the women was 8.1 IU/L and median serum anti-mullerian hormone was 6.3 pmol/L. There was weak positive correlation between age and serum follicle stimulating hormone (*r* = 0.306, *p* < 0.001); weak negative correlation between age and serum anti-mullerian hormone (*r* = -0.48, *p* < 0.001) and very weak correlation between body mass index and serum follicle stimulating hormone (*r *= 0.173, *p* = 0.011). In-vitro fertilization and intra-cytoplasmic sperm injection was the method of fertilization used in 97.4% of the cases and 81.8% of embryos formed were of good quality. Most women (94.5%) had 2 embryos transferred and 89.9% had day-5 embryo transfer done. About 1 in 4 of the women (143/604, 23.7%) had clinical pregnancy and 49.7% of women who got pregnant had delivery of a live baby at term while 11.9% had preterm delivery of a live baby.

**Conclusion:**

Despite increasing use and success of assisted reproductive technology in south-western Nigeria, there is room for improvement in clinical pregnancy rates and live birth rates post- assisted reproductive technology. Complication rates are desirably low.

## Background

Infertility in low- to middle- income countries(LMICs) is a major health problem with consequent negative psycho-social impact especially more on the affected woman. It remains a matter of public health importance that does not get the attention it deserves [1]. The overall prevalence of infertility is estimated at 3.5–16.7% in low- and middle-income countries, with the prevalence as high as 30–40% in some regions of sub-Saharan Africa [2, 3].

The leading causes of infertility in LMICs are male factor and tubal disease secondary to sexually transmitted infections, unsafe abortion and complications of childbirth [4]. Tubal factor infertility has been reported to be as high as 85% in sub-Saharan Africa as against 33% worldwide [2]. Assisted reproductive technology is currently the most effective treatment available for severe male and tubal factor infertility, though expensive and not within the reach of many infertile couples in LMICs [1].

It is encouraging that the number of centres performing assisted reproductive technology in Africa is gradually increasing. However, published reports on large groups of couples accessing ART and its success rates in this region are sparse [5, 6]. Currently there are 69 ART centres in Nigeria registered with the Association for Fertility and Reproductive Health (AFRH) in the country, serving the country’s approximately 250million population. However, published reports on large groups of couples accessing ART and its success rates in this region are sparse [5, 6]. Furthermore, the cost of one IVF cycle in private ART facilities in Nigeria range between 2500 to 3500 United States dollars (USD), which is beyond the reach of the average Nigerian couple and 12,000 to 15000USD in the USA. This study aims to review the characteristics of infertile couple that accessed ART in a public tertiary health centre in south-western Nigeria at a relatively lower cost (ranging from 1000 to 1500USD).

## Materials and methods

This was a retrospective, cross-sectional review, over a ten-year period, of 604 women that accessed artificial reproductive technology (ART) on account of infertility at the Institute of Fertility Medicine, Lagos State University Teaching Hospital, Lagos, Nigeria. Source of referral included the gynaecology clinic of the study institution, public, private, secondary and tertiary health facilities.

The medical records of couples desirous of conception who presented at the study location for assisted conception between January 2012 and January 2022, were reviewed for relevant study data. Women older than fifty-five years seeking ART during the study period were excluded.

Data obtained were age, religion, parity, ethnicity, regularity of menstrual cycle, number of children alive, weight, height, body mass index (BMI), stimulation protocol used, number of cycle attempt at oocyte retrieval, method of fertilization (IVF or IVF/ICSI), quality of embryo used, type of endometrial preparation before transfer, number and grade of embryo transferred, sperm quality, gamete used (use of donor for sperm and/or oocyte) and ART outcome.

Assisted reproductive technology (ART) refers to all treatments or procedures that include the in vitro handling of human oocytes and sperm or embryos for the purpose of establishing a pregnancy. This includes, but is not limited to, in vitro fertilization and embryo transfer, gamete intrafallopian transfer, zygote intrafallopian transfer, tubal embryo transfer, gamete and embryo cryopreservation, oocyte and embryo donation and gestational surrogacy. ART does not include assisted insemination (artificial insemination) using sperm from either a woman’s partner or sperm donor [7].

Agonist long protocol used involves daily administration of GnRH agonists like Buserelin commenced on day twenty-one or a week before the next menstrual flow and continued for four to five weeks. This is down regulation. Then ovarian stimulation with FSH or hMG (75 IU to 250 IU depending on patient characteristics) for twelve to fourteen days. Trans-vaginal ultrasound monitoring of follicular growth is done every three to four days. Ovulation trigger is done at follicular size of 18 mm and above. Egg collection is ensured by thirty-six hours of the ovulation trigger.

Agonist short protocol involves ovarian stimulation commenced on day two of the cycle and commences buserelin from the fourth day of stimulation and may last between twelve to fourteen days. Transvaginal ultrasound for follicular size monitoring is done three to four days. The trigger is also done thirty-six hours before egg collection.

For antagonist protocol, ovarian stimulation with FSH or hMG is started on the second to fifth day of the menstrual cycle and GnRH antagonist (Cetrotide) is commenced when the follicular size is about 14 mm. Trigger is done with Buserelin to stimulate the final follicular maturation prior to oocyte retrieval.

Day-3 embryos were graded using three criteria: (I) Number of cells; (II) Symmetrical arrangements (A—equal size, B – mostly unequal size) and (III) Fragmentation (1—no fragmentation seen, 2—minor/moderate fragmentation, 3—heavy fragmentation).

Embryo grading on day 5 was done using three criteria: (I) Thining/ Expansion of the zona pellucida and liquid cavity (3—early blast, 4—full blastocyst, 5 – hatching, 6—hatched out from the zona); (II) Inner cell mass (ICM) grows into the foetus (A- fully compacted into a ball shape, B- compacted into a cone like shape, C- sparely/no compaction) and (III) Trophectoderm develops into the placenta (A- nicely populated, B- moderately populated, C- sparely populated).

**Fig. 1:**
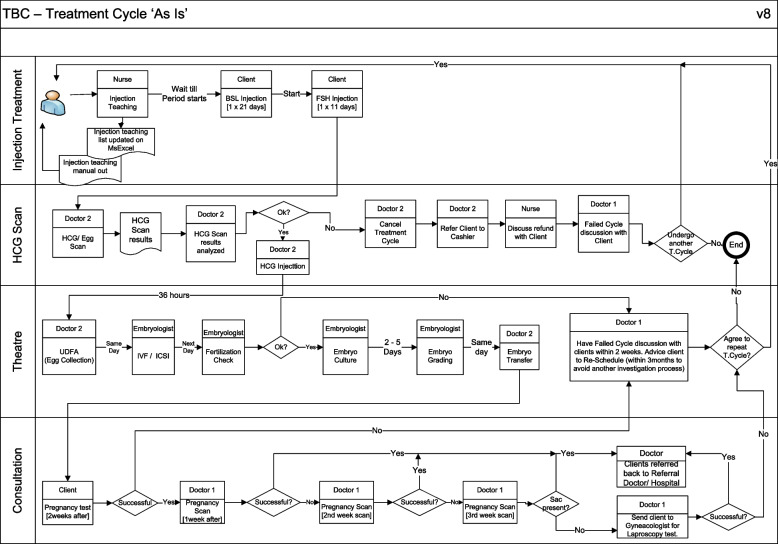
Flow chart of clients eligible for ART (deemed fit after review of baseline results) in the study location

The following embryos were classified as good quality embryo: 3AA, 4AA, 5AA, 6AA, 3AB, 4AB, 5AB, 6AB, 4BA, 5BA, 6BA, 3BB, 4BB, 5BB and 6BB while poor quality embryo included those with grades 4BC, 5BC, 6BC, 4CB, 5CB or 6CB. Clinical pregnancy in this study is defined as evidence of pregnancy by clinical or ultrasound parameters (ultrasound visualization of a gestational sac) [7]. Multiple gestational sacs in one patient are counted as one clinical pregnancy [7]. ART treatment flowchart as done in the study location for clients eligible is depicted in Fig. 1.

The data obtained were entered and analysed using IBM Statistical Product for Service Solutions (SPSS) version 22. Data was expressed in proportions, mean and median. Pearson correlation was used to determine the strength of linear relationship between two continuous variables. For all statistical tests, a confidence level of 95% was used with *p* < 0.05 significance. This study was performed in accordance with the principles stated in the Declaration of Helsinki. Ethical approval for the study was obtained from the Health Research and Ethics Committee of the Lagos State University Teaching Hospital (LASUTH) with protocol number LREC/06/10/1887.

## Results

More than two-third (68.7%) of the women who had ART were aged 35 years and above and the mean age of the women who had IVF or ICSI was of 37.7 ± 6.2 years. Nine in ten (89.7%) of the infertile couples had no previous parous experience and 91.6% had no living child at the time of their presentation for ART (Table 1). The long agonist protocol was the most used stimulation protocol in 93.9% of cases and in 94.0% of the women; oocyte retrieval was achieved during the first cycle of treatment. Oocyte activation was rarely (0.3%) done while IVF/ICSI and IVF were the methods of fertilization used in 97.4% and 2.6% of the cases respectively (Table 1).

**Table 1:** Clinical profile of the women who had ART

Variables	Frequency (*n* = 604)	Percent (%)
**Age group** (years)		
< 35	189	31.3
≥ 35	415	68.7
Mean ± SD 37.7 ± 6.2		
**Parity**		
0	542	89.7
1	48	7.9
≥ 2	14	2.3
**Number of children alive**		
0	553	91.6
1–3	51	8.4
**Stimulation protocol used**		
Agonist short protocol	26	4.3
Antagonist protocol	11	1.8
Long protocol	567	93.9
**Number of cycle attempt at oocyte retrieval**		
1	568	94.0
2	30	5.0
≥ 3	6	1.0
**Egg activation with calcium/ ionophores**		
Yes	2	0.3
No	602	99.7
**Method of fertilization**		
ICSI	588	97.4
IVF	16	2.6

*ICSI* Intracytoplasmic sperm injection, *IVF* In vitro fertilization, *SD* Standard Deviation

For women who accessed ART in this study the mean (SD) and median (IQR) of their body mass index was 27.3 kg/m^2^(4.9) and 27.0 kg/m^2^(24.1–30.5), serum FSH (IU/L) was 10.6(11.4) and 8.1(6.6–10.1), serum AMH (pmol/L)was 17.4(52.5) and 6.3(1.4–17.6), serum estradiol (pmol/L) 28.8(78.2) and 7.4(2.4–10.0) and post ovulation endometrium thickness 8.6 mm(9.2) and 7.5 mm(7.5–9.5) respectively (Table 2).

**Table 2:** Anthropometry and hormonal profile of the women

Variables	Mean ± SD	Median (IQR)
Body mass index(kg/m^2^)	27.3 ± 4.9	27.0(24.1–30.5)
Serum FSH (IU/L)	10.6 ± 11.4	8.1(6.6–10.1)
Serum AMH(pmol/L)	17.4 ± 52.5	6.3(1.4–17.6)
Serum Estradiol (pmol/L)	28.8 ± 78.2	7.4(2.4–10.0)
Post-ovulation endometrium thickness (mm)	8.6 ± 9.2	7.5(7.5–9.5)

*FSH* Follicule stimulating hormone, *AMH* Antimullerian hormone, *SD* Standard Deviation, *IQR* Inter-quartile range

There was weak positive correlation between age and serum FSH (*r* = 0.306, *p* < 0.001); weak negative correlation between age and serum AMH (*r* = -0.48, *p* < 0.001) and very weak correlation between BMI and serum FSH (*r* = 0.173, *p* = 0.011) (Table 3).

**Table 3:** Correlation of clinical, hormonal and sonographic features

Variables	R	*P* value
Age and serum FSH	0.306	< 0.001*
Age and Serum AMH(pmol/L)	-0.479	< 0.001*
BMI and Serum FSH (IU/L)	0.173	0.011*
BMI and Serum AMH(pmol/L)	-0.139	0.148
ET and Age	0.027	0.600
ET and Body mass index(kg/m^2^)	0.072	0.263
ET and Serum FSH (IU/L)	-0.049	0.457
ET and Serum AMH(pmol/L)	-0.021	0.843

*ET* Endometrial thickness, *R* Pearson’s correlation, *FSH* Follicule stimulating hormone, *AMH* Antimullerian hormone

As regards sperm quality, only 27.2% of the male partners had normal seminal fluid parameters while 4.6% had azoospermia. Following fertilization, 81.8% of the embryos formed and used per woman were of good quality. Day-21 down regulation was the most common (92.6%) endometrial preparation done before embryo transfer (Table 2). Only 1 couple (0.2%) had pre-implantation genetic screening done. Most women (94.5%) had 2 embryos transferred and 89.9% had day-5 embryo transfer done (Table 4).

**Table 4:** ART cycle characteristics of the population

Variables	Frequency (*n* = 604)	Percent (%)
Quality of embryo used		
Good	494	81.8
Poor	110	18.2
**Endometrial preparation before transfer**		
Day-2 down regulation	20	3.3
Day-21 down regulation	559	92.6
Not done	2	0.3
Others	23	3.8
**Pre-implantation genetic screening**		
Screened	1	0.2
Not screened	603	99.8
**Number of embryo transferred**		
0	2	0.3
1	24	4.0
2	571	94.5
≥ 3	7	1.2
**Type of embryo transferred**		
Day-3 embryo	59	9.8
Day-5 embryo	543	89.9
Frozen embryo	2	0.3
**Use of hyaluronic acid during embryo transfer**		
Hyaluronic acid used	2	0.3
Hyaluronic acid not used	602	99.7
**Sperm quality**		
Normal	164	27.2
Oligospermia	409	67.7
Azoospermia	28	4.6
Teratozoospermia	3	0.5
**Type of cycle**		
Donor cycle	236	39.1
Patient’s own cycle	368	60.9
**Gamete used**		
Donor	179	29.6
Patient/Self	425	70.4
**Type of donor gamete used**		
Donor egg	161	26.7
Donor embryo	4	0.7
Donor sperm	14	2.3

Less than 1% of the women who had ART developed ovarian hyper stimulation syndrome (OHSS) (0.7%) or ectopic pregnancy (0.3%). About 1 in 4 of the women (143/604, 23.7%) had clinical pregnancy and 49.7% of women who got pregnant had delivery of a live baby at term while 11.9% had preterm delivery of a live baby (Table 5).

**Table 5:** ART complications and outcomes

Variables	Frequency (*n* = 604)	Percent (%)
**Complications**		
OHSS	4	0.7
Abdominal pain	26	4.3
Ovarian torsion	2	0.3
Ectopic pregnancy	2	0.3
Multiple pregnancy	1	0.2
No complication	569	94.2
**Treatment Outcomes**		
Chemical pregnancy	7	1.2
Cycle cancelled	106	17.5
No pregnancy	348	57.6
Clinical pregnancy	143	23.7
**Pregnancy outcomes**	**(*n* = 143)**	**(23.7%)**
Miscarriage	54	37.8
Ectopic pregnancy	2	1.4
Multiple pregnancy	1	0.7
Preterm delivery of live baby	17	11.9
Preterm delivery of still birth	1	0.7
Term delivery of live baby	71	49.7

*OHSS* Ovarian Hyperstimulation Stimulation Syndrome

## Discussion

This is an overview of findings from data of couples who had assisted reproductive technology in a Nigerian teaching hospital over a 10-year period. More than two-third (68.7%) of the women who had ART were aged 35 years and above and the mean age of the women who had IVF or ICSI was of 37.7 years. Orhue et al. in a similar retrospective observational study of 600 couples, who underwent IVF at University of Benin Teaching Hospital over a 5-year period, also reported a mean age of 36.7 ± 4.3 years [8]. Pierce et al [9] noted that the average age of a woman receiving IVF treatment in the United Kingdom was 35 years and comparable to 36 years in the United States [10, 11]. This is similar to observations in our study. A mean age above 35 years seen in women who required ART is in line with the fact that fertility declines with advancing maternal age especially after 35 years of age.

Of these women who had IVF or ICSI done at our Institute of Fertility Medicine, nine in ten (89.7%) of the infertile couples had no previous parous experience and 91.6% had no living child at the time of their presentation for ART. Orhue et al. also noted that most of the women (82.4%) in their study were nulliparas [8]. This suggests that women requiring ART following failure of conventional infertility treatments are largely women with no prior parous experience or living child.

Assessment of male partners in this study revealed that 27.2% had normal seminal fluid parameters, 67.7% had oligospermia and 4.6% had azoospermia. Owolabi et al. in Ile-Ife, Nigeria also found a high rate of abnormal semen quality of male partners of infertile couple with oligozoospermia, teratozoospermia, asthenozoospermia and azoospermia in 25.6%, 18.5%, 11.5% and 6.2% respectively in their study participants [12]. Adewunmi et al. in Lagos, Nigeria reported male factor infertility in 52% of ART seekers [4]. Ugwa et al. in North-western Nigeria alluded to male partners being significant contributors to infertility among infertile couple [13]. However, in their work it was noted that 52.38% of male partners were normospermic while 26.98% and 20.64% were azoospermic and oligospermic, respectively [13]. The relatively larger proportion of normospermia in the study by Ugwa et al. may be explained by their very small sample size.

We observed that though significant, correlation between age and serum FSH was weak and positive while weak negative correlation existed between age and serum AMH. Correlation between BMI and serum FSH was very weak. Our findings on the relationship between these markers of ovarian reserve and age is similar to those of Bozkurt et al. who noted that age positively correlated with basal FSH and inversely correlated with AMH [14]. Age is an important factor associated with the decline in ovarian reserve and diminished ovarian reserve has been documented after the age of 37 [15].

Less than 1% (0.7%) of the women who had ART developed OHSS. Schirmer et al. in the United States noted a current OHSS rate of 0.53% following IVF cycles [16]. Our OHSS rate of 0.7% is similar to that cited by Schirmer et al [16]. However, higher rates of OHSS were recorded in the past. In 2002, Delvigne and Rozenberg published incidences of moderate and severe OHSS estimated at 3%–6% and 0.1%–2%, respectively in patients undergoing in IVF. Our finding supports suggestions that the rates of OHSS are gradually reducing [16, 17]. This is very likely due to many strategies developed and currently employed to reduce the incidence of moderate and severe OHSS in patients undergoing IVF over time.

In this review the ectopic pregnancy rate was 0.3%. Ishihara et al. reported that there was no ectopic pregnancy in treatment outcomes of registered fresh cycles in assisted reproductive technology in Japan in 2017 [18]. In vitro fertilization (IVF) with embryo transfer (ET) has been reported to result in a higher rate of ectopic pregnancies than spontaneous pregnancies [19, 20]. However, our findings and the report by Ishihara et al [18] does not support this as the ectopic pregnancy rate after natural conception is about 2% [21]. The relatively lower ectopic pregnancy rates we found, may be because about 9 in 10 embryo transferred in our study center were done with day-5 embryo. Some studies have shown that decreased uterine contractility during the later luteal phase [22, 23] and blastocyst embryo transfer are associated with reduced rate of ectopic pregnancy compared with cleavage-stage embryo transfer [19, 23]. The low rate (0.3%) of frozen embryo used in this study is because facilities for freezing embryos were not available in the study centre until mid-2022.

About 1 in 4 of the women (143/604, 23.7%) during the period of review had clinical pregnancy and 49.7% of women who got pregnant had delivery of a live baby at term while 11.9% had preterm delivery of a live baby. The pregnancy rate of 23.7% per cycle in this review is comparable to the pregnancy rate per embryo transfer of 23.0% for IVF and 19.2% for ICSI in Japan [18]. The Egyptian IVF registry report in 2005 revealed a pregnancy rate of 35% [24]. Orhue et al [8] in Benin City, Nigeria noted a clinical pregnancy rate of 30% while Makwe et al. in Lagos recorded a similar rate of 33.3% [25]. The higher clinical pregnancy rates in these Nigerian studies may be explained by their relatively smaller sample size, especially in the Lagos study which involved only 24 IVF cycles.

We observed a miscarriage rate of 37.8%, multiple pregnancy rate of 0.7%, preterm live baby rate of 11.9% and term live baby rate of 49.7%. Dimanlig-Cruz et al. in a Canadian population-based study found that crude cumulative incidences of adverse pregnancy and birth outcomes, except for small gestational age, were highest in ART pregnancies [26]. They observed, like some other European population-based studies [27, 28], that “compared with spontaneously conceived pregnancies, ART pregnancies had significantly increased risks of stillbirth (aRR 2.26) multiple birth (aRR 8.95), cesarean delivery (aRR 1.44), preterm birth (aRR 2.06), very preterm birth (aRR 2.99), and low Apgar score (aRR 1.28)” [26]. However, a limitation of our study is that we did not compare pregnancy outcomes in ART and non-ART pregnancies during the study period, to possibly corroborate findings by Dimanlig-Cruz et al. This was because many data of non-ART pregnancy outcomes during the same period were incomplete and/or missing due to their non-electronic paper documentation.

Only 0.3% of our study population had frozen embryo used during their ART. This very low rate was because capacity to freeze embryos was not available until the tail-end of the study period. We noted that Zhu et al [29] had interestingly reported that “the exposure to both frozen sperm and embryo treatment had a complementary effect comparing to the use of fresh sperm and embryos” and associated with increases in the high-quality embryo rate, clinical pregnancy rate, live birth rate and low birth weight rate after the completion of IVF treatment [29]. Based on these we aim to increase the use of cryopreservation during ART in the nearest future in our centre.

## Conclusion

Despite increasing use and success of ART in south-western Nigeria, there is room for improvement in clinical pregnancy rates and live birth rates post-ART. However, complication rates following ART are desirably low.

## Data Availability

The datasets generated and/or analysed during the current study are available in the Mendeley Data repository (doi:10.17632/kkpb82dp3x.1).

## Abbreviations

AMH: Anti-Mullerian Hormone
ART: Artificial Reproductive Technology
BMI: Body Mass Index
CDC: Centre of Disease Control
FSH: Follicle Stimulating Hormone
HFEA: Human Fertilisation and Embryology Authority
hMG: Human Menopausal Gonadotrophin
hCG: Human Chorionic Gonadotrophin
GnRH: Gonadotrophin Releasing Hormone
ICM: Inner Cell Mass
ICSI: Intra-Cytoplasmic Sperm Injection
IQR: Inter Quartile Range
IVF: In-Vitro Fertilization
LASUTH: Lagos State University Teaching Hospital
LMIC: Low-Middle Income Country
LREC: LASUTH Research and Ethics Committee
OHSS: Ovarian Hyper Stimulation Syndrome
SD: Standard Deviation
SPSS: Statistical Package for Social Sciences
RR: Relative Risk
aRR: Adjusted Relative Risk
